# Correlated Eigenvalues of Multi-Soliton Optical Communications

**DOI:** 10.1038/s41598-019-42510-5

**Published:** 2019-04-25

**Authors:** Wen Qi Zhang, Tao Gui, Qun Zhang, Chao Lu, Tanya M. Monro, Terence H. Chan, Alan Pak Tao Lau, V. Shahraam Afshar

**Affiliations:** 1 Laser Physics and Photonic Devices Laboratories, School of Engineering, University of South Australia, Adelaide, Australia; 2 Photonics Research Center, Department of Electrical Engineering, The Hong Kong Polytechnic University, Hong Kong, China; 3 Institute for Telecommunications Research, University of South Australia, Adelaide, Australia; 4 Department of Electronic and Information Engineering, Photonics Research Center, The Hong Kong Polytechnic University, Hong Kong, China; 5 Institute for Photonics and Advanced Sensing, School of Physical Sciences, The University of Adelaide, Adelaide, Australia

**Keywords:** Nonlinear optics, Solitons

## Abstract

There is a fundamental limit on the capacity of fibre optical communication system (Shannon Limit). This limit can be potentially overcome via using Nonlinear Frequency Division Multiplexing. Dealing with noises in these systems is one of the most critical parts in implementing a practical system. In this paper, we discover and characterize the correlations among the NFT channels. It is demonstrated that the correlation is universal (i.e., independent of types of system noises) and can be exploited to maximize transmission throughput. We propose and experimentally confirm a noise model showing that end-to-end noise can be modelled as the accumulation of noise associated with each segment of optical communication which can be dealt with independently. Also, each point noise can be further decomposed into different components, some of which are more significant (and even dominating) than others. Hence, one can further approximate and simplify the noise model by focusing on the significant component.

## Introduction

Data traffic has been growing at a rate of more than 60% per year^1^. Such astronomical growth has sparked an urgent need to significantly increase the network transmission capacity, posing a critical technical challenge for system designers.

One main fundamental challenge to further enhance data transmission bandwidth is to manage fibre nonlinearities^2^.

Signal propagation across an optical fibre is governed by the nonlinear Schrödinger equation. The channel is nonlinear, unlike other typical transmission media such as copper wires and radio waves^2^. Traditionally, fibre nonlinearities are often regarded as channel impairments, and hence should be eliminated or mitigated. Instead of dealing with fibre nonlinearities directly, existing schemes are often based on a “flawed” approach in that they apply “off-the-shelf” methods originally developed for classical linear time-invariant radio frequency channels (typically with additive white Gaussian noise). This approach ignores the detail of the underlying fibre physics, and attempts to draw loose analogies between macroscopic channel impairments (e.g. dispersion caused by a linear multipath channel) encountered in microwave channels with those in optical channels (e.g. dispersion due to wavelength-dependent refractive index, fibre geometry or nonlinearities). In essence, nonlinearities are assumed to be weak and hence can be treated and suppressed as small perturbations^3,4^.

The underlying premise behind this perspective is that signals are processed often in the time domain and/or the (linear) frequency domain (where signals are obtained by applying linear transformation such as Fourier transform on the time-domain signals). However, fibre nonlinearities cannot be completely eliminated by invoking these linear signal processing techniques, leading to undesirable inter-symbol interference (ISI) and inter-channel interference (ICI)^4^. As a result, it was noted that fibre nonlinearities can impose a fundamental limit (known as linear Shannon limit) on the data transmission capacity^3^.

A different paradigm to the problem has received a lot of attention in the past few years. In this paradigm, fibre nonlinearity and dispersion effect are merely seen as ordinary physical characteristics needed to be managed directly, rather than simply evading them as disadvantages^5–10^. In particular, their approaches are based on the use of nonlinear Fourier transform (NFT), or direct scattering transform^11–13^. Higher order dispersion effects are often ignored, and the linear loss term is assumed to be perfectly compensated by the distributed Raman amplification (DRA).

Mathematically, the NFT provides a systematic method for solving the class of integrable nonlinear Schrödinger equation, whereas, in engineering perspective, NFT can “decompose” the nonlinear fibre channel into multiple independent subchannels in the (nonlinear) spectral domain. To a great extent, it mirrors the widely used wavelength-division multiplexing (WDM), a technology which multiplexes multiple optical carrier signals and transmitted in a single optical fibre. The fundamental difference between WDM and NFT based approach is in how the “*modes*” or “*subchannels*” are defined.

Roughly speaking, WDM employs Fourier Transform (FT) such that each wavelength (or its corresponding linear frequency) is essentially a “transmission mode”. When signal-to-noise ratio (SNR) is low and nonlinearities are not severe, interference among these modes are negligible. However, as data rate (and also signal power) increases, nonlinearities become significant and the transmission modes defined by FT can now significantly interfere with each other. This significantly limits the performance of the fibre-optic communications systems, especially in long-haul transmissions. In^6^, NFT was used instead of FT, such that the resulting nonlinear normal modes will not interfere with each other even in the presence of nonlinear effects.

This idea of decoupling a nonlinear channel into multiple independent subchannels plays the central role in NFT based communications. As a result of the channel decomposition, one can separately design communications for each individual subchannel, and hence greatly reduce the system complexity. Also, inter-channel interference is eliminated with a proper allocation of the (nonlinear) spectrum to users at least in the noise-free scenario. This scheme is called nonlinear frequency division multiplexing (NFDM)^6^.

The development of NFT-based transmission systems is only in its infancy stage at the moment. Some preliminary experimental works have already been done to demonstrate the concepts. The spectrum of a time domain signal, after applying the NFT, is composed by discrete and continuous spectrum. Both continuous^6,14–17^ and discrete^18–23^ spectra have been proposed for optical transmission systems. Using a discrete 1-, 2-, 3-eigenvalue configuration together with on-off keying, Dong *et al*.^18^, have achieved 1.5 Gbps transmission over 1800 km. In^24^ a two-eigenvalue signal together with QPSK modulation has been used to achieve 4 Gbps transmission rate over 640 km, followed by QPSK on 7 discrete eigenvalues achieving 14 Gbps transmission rate^25^. 16 QAM modulation on single discrete eigenvalue is then reported to upgrade the data rate to 24 Gbps^26^. On the other hand, experimental studies of pure continuous spectral modulation have recently been demonstrated by Le *et al*.^17,27–29^ and most recently a demonstration of joint continuous and discrete spectrum modulation is reported^30^. Particularly^29^, demonstrated that the continuous spectrum based NFDM system have 1 dB performance advantage over the conventional OFDM transmission with 32 Gbps transmission rate over 1464 km.

As mentioned above, NFT based methods lead to channel decomposition with zero inter-channel interference. Unfortunately, perfect channel decomposition is only theoretically possible in the absence of noise. In practice, noise can be generated in the transmitter and the receiver (e.g., quantization, clipping), and also during propagation in the fibre (e.g., due to in-line or point amplification). These noises will induce correlations among individual subchannels, affecting the capacity of the transmission system. Despite the crucial role of noise in determining the actual capacity of an NFT-based transmission system, effects of noise on NFT continuous and discrete spectra have only been studied in limited cases. Zhang *et al*.^31^, have studied the effect of propagation noise (with Gaussian distribution) on the spectral amplitudes and the discrete eigenvalue of the channel output when the input is a fundamental soliton. Correlation and signal-dependent noise have been considered in^1,30,32^. Derevyanko *et al*.^1^, have developed an approximated noise (with Gaussian distribution) model for continuum spectra NFT based transmission and estimated a lower bound for the capacity. Based on their model, they find the noise properties of NFT continuous spectra after propagating through a fibre in presence of noise. The statistics of scattering vectors due to noise has been considered in^33^ and investigating the covariance of discrete eigenvalues has been identified as an interesting problem in NFT. In^34^, transmission of second order solitons with QPSK-modulated discrete spectral amplitude have been studied and the statistic of amplitude phase variation has been compared with those of eigenvalues. The effect of noise on phase-modulated signals (with continuous and discrete spectrum) have been considered in^30^, and it has been concluded that encoding more bits on discrete part seems to be feasible.

In this paper, we investigate–both experimentally and through simulation–the noise properties of optical communications systems based on input optical pulses with discrete NFT eigenvalues (multi-soliton). In particular, we consider second and third order solitons, with only two and three discrete eigenvalues, respectively, and square pulses with both discrete and continuous eigenvalues. There are four main aspects of our contributions:

1. Unlike in the case of fundamental solitons, analysis of eigenvalue perturbation for multi-soliton or even for general signals is very limited. In this paper (Section 2), We demonstrate that the discrete eigenvalues of a multi-soliton signal propagating through the network are correlated, regardless of the different types of noise that have been introduced at different stages of signal preparation, propagation, and detection. We also show that such correlation properties can be used to maximize the transmission throughput so that input signal constellation can be optimized to support high data transmission rate. In addition, we also show that the correlation between discrete eigenvalues depends on what we define as the *nonlinear phase*.
2. In order to explain perturbation properties of eigenvalues, particularly the correlation among discrete eigenvalues, we propose a model (Section 4.1 and 4.2) for discrete eigenvalue perturbation such that the perturbation of eigenvalues can be written as the sum of individual eigenvalue perturbations caused by the injection of the noise in each fibre segment.
3. We show that the noise effects do not accumulate and noise associated with each segment of optical communication can be dealt with independently. The innovative aspect of our model is the modelling assumption that the individual eigenvalue perturbations are essentially insensitive to the noises injected before or after the segments. As a result, these small individual eigenvalue perturbations can be modelled as independently distributed. As an important consequence of this, we show both experimentally and numerically that the angle of the principal eigenvector of the covariance matrix is related to nonlinear phase difference. We are not aware that this model has been considered in existing literature.
4. We identify and demonstrate that the effect of noise can be decomposed into different components and show that the noise components along the signal have more dominating effect than others. This provides more insights into the reason behind the eigenvalues correlation and also suggests that one can further approximate and simplify noise effects by focusing on those dominating noise components.

## Basic Principle

The noisy signal evolution across an optical fibre is often modelled as the following stochastic nonlinear Schrödinger equation (SNLSE)

1 $$\frac{\partial A(s,l)}{\partial l}-\frac{j{\beta }_{2}}{2}\frac{{\partial }^{2}A(s,l)}{\partial {s}^{2}}=-\,j\gamma |A(s,l{)|}^{2}A(s,l)+j\kappa N(s,l),\,0\le l\le  {\mathcal L} \,{\rm{km}},$$

where $j=\sqrt{-1}$. The function *A*(*s*, *l*) is the complex envelope of the signal propagating along the fibre. The parameter *β*_2_ is the group velocity dispersion (GVD) coefficient. The GVD coefficient for silica fibres is *β*_2_ = −2 × 10^−23^ s ^2^ km^−1^ when the input wavelength is 1.55 μm. The parameter *γ* is the nonlinear coefficient. The positive real number ${\mathcal L}$ denotes the length of the optical fiber. The term *jκN*(*τ*, *l*) represents the optical noise field, which could be modelled as a zero mean circularly symmetric complex white Gaussian noise process^35,36^ with

2 $${\rm{E}}[N(s,l){N}^{\ast }(s^{\prime} ,l^{\prime} )]=\delta (s-s^{\prime} )\delta (l-l^{\prime} ),$$

where we use “*” to denote the complex conjugate, and *δ*(*x*) means Dirac delta function, and *κ* is a coefficient that determines the strength of the noise.

After applying the following variable transformations

3 $$q=\frac{A}{\sqrt{P}},\,\,t=\frac{s}{T},\,\,z=\frac{l}{ {\mathcal L} },$$

where

4 $$P=\frac{2}{\gamma  {\mathcal L} },\,\,T=\sqrt{\frac{|{\beta }_{2}| {\mathcal L} }{2}},$$

we obtain the normalised SNLSE

5 $$j{q}_{z}(t,z)={q}_{tt}(t,z)+2|q(t,z){|}^{2}q(t,z)+j\varepsilon G(t,z),$$

where the noise *εG*(*t*, *z*) is a zero mean circularly symmetric complex white Gaussian noise with power spectral density ${\varepsilon }^{2}=\frac{\gamma }{\sqrt{2|{\beta }_{2}|}}{\kappa }^{2}$.

Under the model (5), the effect of noise in soliton parameters was studied. In particular, the statistics of the eigenvalue was reported in^37,38^, and the arrival time jitter, namely Gordon-Haus effect, was studied in the celebrated paper^39^. The research about soliton transmission control, regarding the issue of timing jitter, can be found in^40–43^. The Gordon-Mollenauer effect, referring to the soliton phase jitter, was investigated in^44^, and the work about its statistics in solitonic dispersion phase shift keying systems was studied in^45–47^.

Let *L* be an operator on *q*(*t*, *z*) where

6 $$L=j\,(\begin{array}{cc}\frac{\partial }{\partial t} & -q(t,z)\\ -{q}^{\ast }(t,z) & -\frac{\partial }{\partial t}\end{array}),$$

The eigenvalues of the operator *L* are invariant in *z* as the signal *q*(*t*, *z*) propagates through the fibre. In the definition of the NFT, we suppress the variable *z* because it is only useful when we need to derive the spatial signal propagation through an optical fibre. Throughout this paper, we assume that $q(t)\in {L}^{1}({\mathbb{R}})$ and *q*(*t*) → 0, *t* → ∞.

The NFT of a signal *q*(*t*) is defined via the spectral analysis of the operator *L*. Specifically, we need to solve the eigenvalue problem

$$Lv=\lambda v$$

at first, which is equivalent to the ordinary differential equation (ODE)

7 $${v}_{t}=(\begin{array}{cc}-j\lambda  & q(t)\\ -{q}^{\ast }(t) & j\lambda \end{array})v$$

called the scattering problem. Using boundary conditions

8 $$\mathop{\mathrm{lim}}\limits_{t\to -\infty }|v(t,\lambda )-(\begin{array}{c}1\\ 0\end{array}){e}^{-j\lambda t}|=0,$$

we obtain a solution of (7).

Let *v*(*t*, *λ*) = [*v*_1_(*t*, *λ*), *v*_2_(*t*, *λ*)]^Τ^. The coefficients *a*(*λ*) and *b*(*λ*) are called scattering data, which can be obtained by calculating

9 $$a(\lambda )=\mathop{\mathrm{lim}}\limits_{t\to \infty }{v}_{1}(t,\lambda ){e}^{j\lambda t},$$

and

10 $$b(\lambda )=\mathop{\mathrm{lim}}\limits_{t\to \infty }{v}_{2}(t,\lambda ){e}^{-j\lambda t}.$$

The nonlinear Fourier transform of a function *q*(*t*) is defined with the help of the scattering data. The NFT of a signal *q*(*t*) is composed of its spectrum and the corresponding spectral amplitudes. The spectrum is composed of the discrete and continuous spectrum. The discrete spectrum is a set of isolated complex points called (discrete) eigenvalues, which are zeros of the scattering data *a*(*λ*) on the upper half complex plane ${{\mathbb{C}}}^{+}\triangleq \{c\in {\mathbb{C}}:\,{\rm{Im}}(c)\, > \,0\}$. The continuous spectrum is the real line ${\mathbb{R}}$. The corresponding spectral amplitudes are defined as follows. The discrete spectral amplitude subject to an eigenvalue ${\lambda }_{k}\in {{\mathbb{C}}}^{+}$ is

11 $${Q}^{(d)}({\lambda }_{k})=\frac{b({\lambda }_{k})}{{a}^{\text{'}}({\lambda }_{k})},\,\,k=1,2,\ldots ,N,$$

where ${a^{\prime} ({\lambda }_{k})\triangleq \frac{{\rm{d}}a(\lambda )}{{\rm{d}}\lambda }|}_{\lambda ={\lambda }_{k}}$, and *N* is the number of the zeros of *a*(*λ*). The continuous spectral amplitude is defined as

12 $${Q}^{(c)}(\lambda )=\frac{b(\lambda )}{a(\lambda )},$$

where $$\lambda \in {\mathbb{R}}$$.

It is well known that the spectrum of the signal keeps invariant as a signal propagates through an optical fibre in the noise-free case. The spatial evolution of the spectral amplitudes are summarised as follows:

13 $${Q}^{(c)}(\lambda ,z)={Q}^{(c)}(\lambda ,0){e}^{-4j{\lambda }^{2}z},$$

and

14 $${Q}^{(d)}({\lambda }_{k},z)={Q}^{(d)}({\lambda }_{k},0){e}^{-4j{\lambda }_{k}^{2}z},\,\,k=1,2,\ldots ,N,$$

where *Q*^(*c*)^(*λ*, *z*) and *Q*^(*d*)^(*λ*_*k*_, *z*) are respectively a continuous and a discrete spectral amplitude at position *z*, and *z* > 0, and $-4j{\lambda }_{k}^{2}$ is the *NFT channel gain coefficient* and ${e}^{-4j{\lambda }_{k}^{2}z}$ as the channel gain. Here, we also define the phase of *Q*^(*c*)^(*λ*, *z*) and *Q*^(*d*)^(*λ*_*k*_, *z*) as *nonlinear phase*.

## Results: Eigenvalue correlation

Pulse propagation experiments and simulations were carried out using pulses with only two discrete eignevlues ranging from *λ*_1_ = 0.3 *j* to 0.75 *j* and *λ*_2_ = 0.9 *j* to 1.35 *j* in steps of 0.15 *j*.

**Figure 1:**
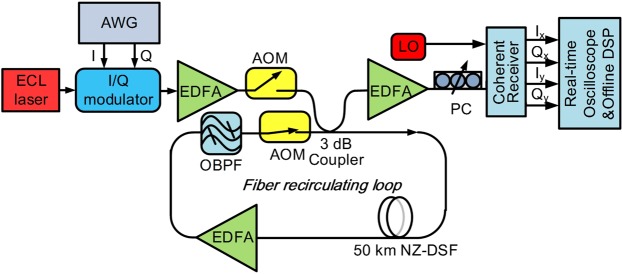
Experimental setup. ECL: external cavity laser; AWG: arbitrary waveform generator; AOM: acousto-optic modulator; EDFA: erbium doped fiber amplifier; NZ-DSF: non zero dispersion shifted fiber; OBPF: Optical band pass filter; LO: local oscillator; PC: polarization controller.

Figure 1 shows the experimental setup for the eigenvalue correlation transmission system. The transmitter (TX) comprises a 92 GSa/s arbitrary waveform generator (AWG) providing a drive signal for an IQ modulator which generates 1 GBd optical soliton pulses train in a single polarization. The outputs of the modulator are amplified and launched into a recirculating fibre loop. Since the theory of NFT is based on the integrability property of the lossless nonlinear Schrödinger equation, a short span of 50 km NZ-DSF fiber (with *α* = 0.19 *dB*/*km*, *β*_2_ = −5.01 *ps*^2^*km*^−1^ and *γ* = 1.2 *W*^−1^*km*^−1^) has been considered in the recirculating loop to minimize perturbation of signal power evolution along the link. An EDFA is placed after the fibre to compensate the span loss and ensure the same launched power after each loop. A flat-top optical filter with a 3 dB bandwidth of 1 nm is used inside the loop to suppress the out-of-band amplified spontaneous emission (ASE) noise. At the receiver, the signal is first aligned in a particular polarisation by a polarisation controller and then detected by an integrated coherent receiver. The output electrical waveforms are sampled by a digital storage scope (with a sampling rate of 80 GSa/s and a bandwidth of 33 GHz) followed by off-line digital signal processing (DSP).

**Figure 2:**
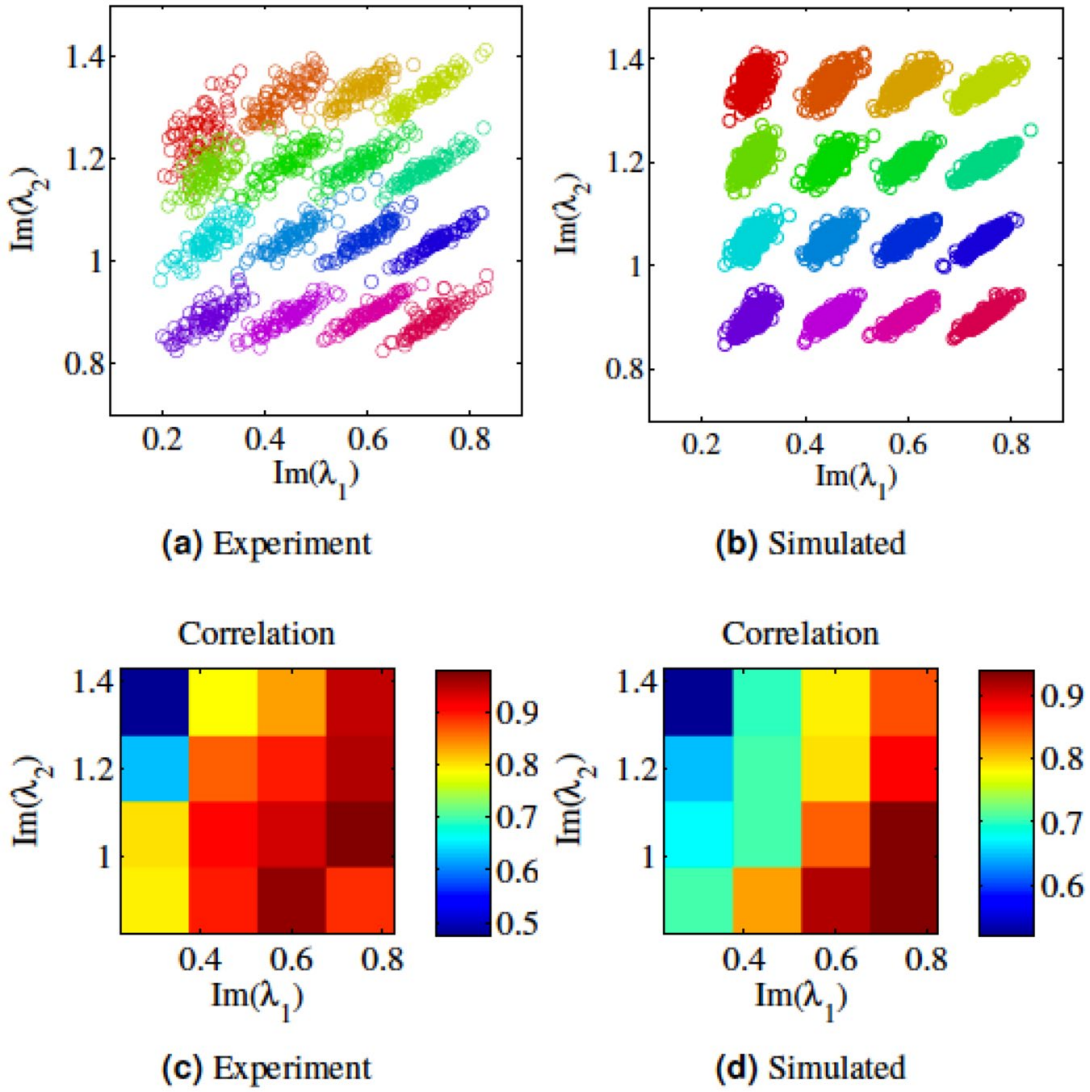
Experimental (**a**), and simulated (**b**) distribution of eigenvalues after 400 km transmission. The different colours are used to help distinguish different input eigenvalue pairs. (**c**) and (**d**), experimental and simulated contour plot of correlation coefficient, respectively.

Figure 2 shows the experimental and simulated received signal distributions of the group of 2-soliton pulses (with different set of *λ*_1_ and *λ*_2_, |*Q*| = 1 and initial nonlinear phase, defined in Section 2, of zero) after propagating a distance of 400 km, equivalent of 8 times circulation within the fiber loop. For each pulse set (*λ*_1_, *λ*_2_) simulations were run for 500 times, with noiseless input pulses but distributed random noise within the fibre. After propagation, the eigenvalues of these 500 outputs were then calculated using a forward difference method^6^, see Simulation section in Methods 5.1. Figure 2a,b show the experimental and simulation distribution of each set of eigenvalues after propagation, respectively. A circular distribution of the two eigenvalues are expected for totally uncorrelated eigenvalues. However, the results show a linear-like distribution, which indicates a positive correlation. To quantify this, we have calculated the sample correlation coefficient between *Im*(*λ*_1_) and *Im*(*λ*_2_) for each set of eigenvalues and represented them in Fig. 2c,d, for experimental and simulation results, respectively. Note that the sample correlation between *n* samples of (*x*_*i*_, *y*_*i*_) for *i* = 1, …, *n* is defined as

$$\frac{\sum _{i=1}^{n}({x}_{i}-\bar{x})({y}_{i}-\bar{y})}{(n-1){s}_{x}{s}_{y}}$$

where (1) $\bar{x}$, $\bar{y}$ are sample means of *x* and *y*, and (2) *s*_*x*_ and *s*_*y*_ are the sample standard deviations of *x* and *y*.

Both experimental and simulation results show the following common characteristics that indicate the correlated nature of NFT eigenvalues:

1. Both experiment and simulation show positive correlation represented by elongated elliptical shapes with a slightly different orientation of major axis. From a signal design point of view, and as we demonstrated through a simulation below, one can appropriately leverage such correlation properties by using more compact arrangment of NFT eigenvalues in the direction of the minor axis of the elliptical distribution and achieve a higher spectral efficiency.
2. In both cases, correlation decreases as *λ*_2_ increases or *λ*_1_ decreases.

The angle of the scattering ellipsis’ orientation in Fig. 2(a,b) is partially related to *λ*_1_, *λ*_2_ as well as propagation distance and nonlinear phase. The details of such relations are provided in Section 4.2.

Some discrepancies between experimental and simulation results are also observed. Qualitatively, the orientation of the distributions of the two eigenvalues and their correlation are different for experiment and simulations results. This could be explained by noting that in simulations, we only consider white Gaussian noise added during the propagation along the fibre. In the experiment, however, apart from the noise generated during propagation, noises are also induced during the pulse generation stage (e.g., when using the AWG to generate the electrical signals and the IQ modulator to generate optical signals) and detection stages. In general, different types of noise can affect a signal at different stages of generation, propagation, and detection. In Supplementary Material 1, we show the simulations of the different effects of different types of noise. The results demonstrated that correlations between eigenvalues of a signal commonly exist.

**Figure 3:**
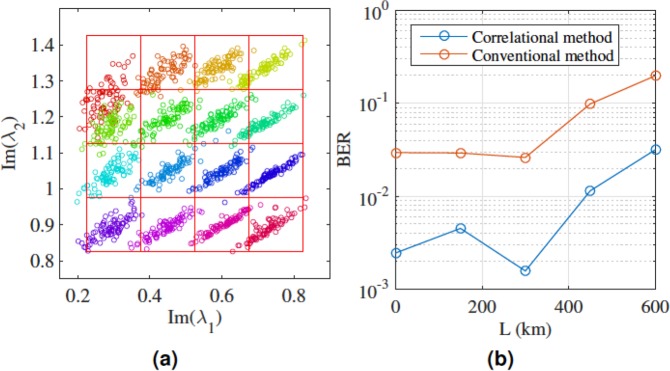
Distribution of eigenvalues for constellation of 2-soliton pulses after propagating for 0.1 normalized distance. (**a**) Conventional decoding method of defining a grid over the constellation chart from the experimental data set. (**b**) Bit error rate (BER) calculated using both the conventional method and the correlational method for different propagation length.

Correlations of NFT eigenvalues has a significant impact on designing an NFT-based fibre optic network. In Supplementary Material 4, we consider an example illustrating how to exploit such correlations to improve the performance of a system. The concept also does great help to the symbol decision. An example of utilising the correlation to improve BER is shown below using the experimental dataset (Fig. 2(a)). Figure 3(a) shows a grid overlay on top of the constellation that is used to decode the signal conventionally. The vertices of the grids are 0.225, 0.375, 0.525, 0.675 and 0.825 for the x-axis and 0.825, 0.975, 1.125, 1.275 and 1.425 for the y-axis. A symbol is decoded correctly if it falls into its corresponding grid. However, if assuming the noise are in correlated 2-D Gaussian distribution, one can apply the covariance matrix to normalise the Euclidean distance by using the following equation to decode the signal:

$$\mathop{{\bf{argmax}}}\limits_{i=1,\mathrm{...},16}\{\exp (\,-\,(S-{U}_{i}){C}_{i}^{-1}{(S-Ui)}^{T})\}$$

where *S* = (**x**_***i***_, **y**_***i***_)|_*i*=1, …, 16_ denotes the received (*Im*(*λ*_1_), *Im*(*λ*_2_)) which has 16 different possibilities. *C*_*i*_ is calculated as

$${C}_{n}=[\begin{array}{cc}cov({{\bf{x}}}_{{\bf{i}}},{{\bf{x}}}_{{\bf{i}}}) & cov({{\bf{x}}}_{{\bf{i}}},{{\bf{y}}}_{{\bf{i}}})\\ cov({{\bf{y}}}_{{\bf{i}}},{{\bf{x}}}_{{\bf{i}}}) & cov({{\bf{y}}}_{{\bf{i}}},{{\bf{y}}}_{{\bf{i}}})\end{array}],i=1,2,\mathrm{...},16$$

where *cov*(⋅) denotes the covariance matrix. *U*_*i*_ is the variance of *S* which can be calculated as:

$${U}_{i}=({\bf{E}}({{\bf{x}}}_{{\bf{i}}}),{\bf{E}}({{\bf{y}}}_{{\bf{i}}})),$$

where **E**(⋅) denotes expectation.

The BER of the two decode method is plotted in Fig. 3(b). By taking advantage of the correlation, BER of the same data set is 10 fold smaller than the conventional method.

**Remark**: The above example is used only to demonstrate how correlations can be exploited to increase data rate.

**Figure 4:**
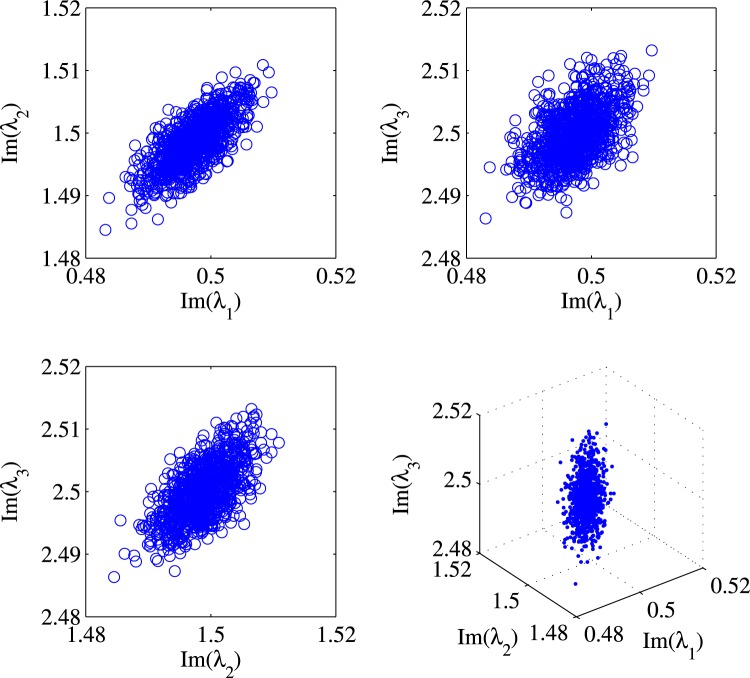
Correlations between all possible pairs of eigenvalues for a 3-eigenvalue input signal. The bottom right figure shows correlation of all eigenvalues in 3 dimensional parameter space.

Further simulation studies reveal that the correlation exists in systems with larger number of discrete eigenvalues, e.g. 3. Figure 4 shows a case with 3 discrete eigenvalues 0.5 *j*, 1.5 *j* and 2.5 *j*. The subplots of Fig. 4 show the correlation between eigenvalues *λ*_1_ and *λ*_2_, *λ*_1_ and *λ*_3_, *λ*_2_ and *λ*_3_ as well as a distribution of the eigenvalues in a 3-D parameter space. Similar correlations can also be found in systems with even more eigenvalues.

One fundamental challenge in designing eigenvalue communications system is to characterise the noise in the discrete eigenvalues. In the previous section, we have discussed the correlation of discrete eigenvalues for short distance (of normalised length 0.1) experimentally and numerically. To interpret or explain such correlation among eigenvalues, we propose a model for eigenvalue perturbation and examine how additive noises affect the eigenvalues in the following section. Based on the model, we observe that the eigenvalues are significantly affected by the same “noise component” which matches the signal itself. As a result, the perturbation of the eigenvalues can be greatly correlated.

To better illustrate the idea, consider a simple additive noise model:

$$y(t)=x(t)+n(t)$$

where x(t) is a signal in the time domain and *n*(*t*) is an additive white Gaussian noise. If we look at two different linear frequencies *f*_1_ and *f*_2_, let *Y*(*f*), *X*(*f*) and *N*(*f*) be the ordinary (linear) Fourier Transform of *y*(*t*), *x*(*t*) and *n*(*t*), then *Y*(*f*_1_) − *X*(*f*_1_) = *N*(*f*_1_) and *Y*(*f*_2_) − *X*(*f*_2_) = *N*(*f*_2_). The interesting fact is that *N*(*f*_1_) and *N*(*f*_2_) are independent of each other (corresponding to two independent harmonic components of the white noise *n*(*t*)). In other words, the harmonic noise component at frequency *f*_1_ will have no effects on the perturbation *Y*(*f*_2_) − *X*(*f*_2_). Similar is true for the harmonic noise component at frequency *f*_2_ on the perturbation *Y*(*f*_1_) − *X*(*f*_1_).

However, this is no longer the case for the eigenvalues of *y*(*t*) in the NFT domain. In the following section, we consider a model for eigenvalue perturbation, using which our simulation shows that the eigenvalue perturbation is greatly affected by a noise component that matches the signal *x*(*t*). As a result, the eigenvalue perturbation becomes correlated.

## Results: Modeling eigenvalues perturbation

In the following, we will develop a full model of eigenvalue perturbation, based on our two observations on properties of NFT discrete eigenvalue perturbation in an optical communication system:

1. **Split Step Method for noise;** deterministic (due to nonlinearity and dispersion) and stochastic (e.g., due to all signal amplification) noise effects can be separated, in a similar fashion as linear and nonlinear processes in Split Step Method, and
2. **No noise hysteresis;**, the effect of noise associated with each segment of optical communication can be dealt with independently.

Note that the Split-Step Method is a classical technique to numerically evaluate how a signal propagates along a fibre. Its central idea is to divide the fibre into many consecutive segments such that one can “separately” model and consider the effects of different channel impairments (i.e., fibre nonlinearity, chromatic dispersion and noise). Our eigenvalue model is based on the same methodology. However, as the fibre nonlinearity and the chromatic dispersion as a whole will not perturb the eigenvalues (the beauty and also the fundamental property of NFT), we only need to consider the effect of additive noise on eigenvalue perturbation. A key advantage of our model is that we can now model the eigenvalue perturbation as an accumulated sum of many smaller perturbations introduced in each fibre segment. Furthermore, we observe and model that these perturbations are essentially independent, and hence leading to a much simpler model.

**Figure 5:**
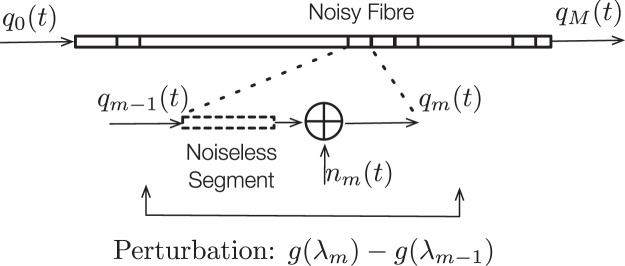
Channel Model: Fiber is considered as a concatenation of *M* segments. The perturbation of eigenvalues (or their function values) is modelled as the accumulation of many perturbations caused by the addition of noises in each segment. Each perturbation *ε*_*m*_ is further modelled as independent, depending only on the deterministically distorted signal ${\bar{q}}_{m}(t)$.t

First, we will state the framework based on which the eigenvalue noise perturbation model is developed. When a signal propagates along a fibre, it will be distorted by various channel impairments such as noises and fibre nonlinearity and dispersion, causing its shape to change during propagation. In this paper, we will assume that the fibre loss can be perfectly compensated by inline distributed amplification. To model the process, we treat a fibre as a concatenation of many short fibre segments. As each segment is short, fibre nonlinearity and noise can often be modelled separately. Under this model, when a signal propagates in a segment, it will undergo two phases. In the first phase, it will be distorted by fibre nonlinearity, assuming that the segment is noiseless. Then in the next phase, a white Gaussian noise (whose power is proportional to the length of the fibre segment) will be added. The resulting signal will then become the input of the next segment, and the same process will continue until it reaches the end of the fibre, see Fig. 5.

Modelling a fibre as a concatenation of short segments is only the first step. Due to fibre nonlinearities and the coupling effects between noises and signals, it is still challenging to derive an analytic model to characterise the eigenvalues perturbations. In the following, we aim to simplify the model.

### Simplification 1: Noise Decoupling

One of the challenges in deriving a model for characterising the perturbation of discrete eigenvalues is due to the coupling effects of stochastic signal dependent noises (e.g., due to inline amplification) and the distortion due to nonlinearities and dispersions. In the following, we propose a new simplification paradigm to decouple the two noise effects. Our proposed simplification is based on the observation that *perturbation of eigenvalues at the end of the fibre can be accurately modelled by “summing up” all the small perturbations of eigenvalues in the segments*.

Suppose the input to the fibre is *q*_0_(*t*). Divide the fibre into *M* segments and let *q*_*m*_(*t*) be the output of the signal after propagating *m* segments (or equivalently the input to the (*m* + 1)^*th*^ segment). Define Λ_*m*_ as the set of discrete eigenvalues of *q*_*m*_(*t*). Let *g*(Λ_*m*_) be a (scalar or vector valued) function of Λ_*m*_ of interest.

Notice that

$$g({{\rm{\Lambda }}}_{M})-g({{\rm{\Lambda }}}_{0})=\sum _{m=1}^{M}\,g({{\rm{\Lambda }}}_{m})-g({{\rm{\Lambda }}}_{m-1}).$$

Therefore, we can characterise the perturbation *g*(Λ_*M*_) − *g*(Λ_0_) by characterising the perturbation *g*(Λ_*m*_) − *g*(Λ_*m*−1_) for all *m*, see Fig. 5 and Table 1.

**Table 1:** Notations.

Symbol	Definition
*q*_*m*_(*t*)	output of signal after propagating *m* segment
${\bar{q}}_{m}(t)$	output of signal after propagating *m* segment, assuming no added stochastic noises
*n*_*m*_(*t*)	noises added in the *m* segment
${\hat{q}}_{m}(t)$	${\bar{q}}_{m}(t)$ + *n*_*m*_(*t*)
Λ_*m*_	discrete eigen-values of *q*_*m*_(*t*)
${\hat{{\rm{\Lambda }}}}_{m}$	discrete eigen-values of ${\hat{q}}_{m}(t)$
*g*(Λ)	a function of Λ

Observing each individual term, the perturbation *g*(Λ_*m*_) − *g*(Λ_*m*−1_) is caused by the injection of a noise at the *m*^*th*^ segment. First, we want to point out that the perturbation depends on both the injected noise *n*_*m*_(*t*) and the input of the segment *q*_*m*−1_(*t*) (which in turn also depends on the noises added in the previous segments). However, we claim that; *the influence due to the noise added in the previous segments are insignificant (and hence can be ignored)*.

Let ${\bar{q}}_{m}(t)$ be the output of the signal after propagating through *m*^*th*^ segments, assuming there are no noises. Hence, ${\bar{q}}_{m}(t)$ is a deterministic signal. Due to fibre nonlinearity, its shape will vary with *m*. However, the discrete eigenvalues for all ${\bar{q}}_{m}(t)$ remained unchanged. Let *n*_*m*_(*t*) be the noise added in the *m* segments,

15 $${\hat{q}}_{m}(t)={\bar{q}}_{m}(t)+{n}_{m}(t)$$

and ${\hat{{\rm{\Lambda }}}}_{m}$ be its set of discrete eigenvalues. In other words, ${\hat{q}}_{m}$ is obtained by propagating *q*_0_(*t*) noiselessly across *m* segments, followed by the addition of the noise *n*_*m*_(*t*). See Fig. 5. We claim that $g({\hat{{\rm{\Lambda }}}}_{m})-g({{\rm{\Lambda }}}_{0})$ is in indeed a good approximation for *g*(Λ_*m*_) − *g*(Λ_*m*−1_).

Let ${\varepsilon }_{m}=g({\hat{{\rm{\Lambda }}}}_{m})-g({{\rm{\Lambda }}}_{0})$, then $g({{\rm{\Lambda }}}_{M})-g({{\rm{\Lambda }}}_{0})\approx {\sum }_{m\mathrm{=1}}^{M}\,{\varepsilon }_{m}$ or equivalently,

16 $$g({{\rm{\Lambda }}}_{M})\approx g({{\rm{\Lambda }}}_{0})+\sum _{m=1}^{M}\,{\varepsilon }_{m}\mathrm{.}$$

#### Benefits

The above approximation points out that the end-to-end perturbation is now modelled as the sum of a collection of independently distributed local perturbations (as ${\bar{q}}_{m}$ is deterministic and the noise *n*_*m*_(*t*) is independently distributed for all *m*). The main benefit of the model is its simplicity, decoupling the stochastic noises (caused by inline amplification) from the deterministic dispersion and nonlinearities.

To be more precise, we have already seen that the local perturbation of the eigenvalues

$$g({\hat{{\rm{\Lambda }}}}_{m})-g({{\rm{\Lambda }}}_{0})$$

depends on *m* (and more precisely the signal that enters the *m* fibre segment, i.e., ${\bar{q}}_{m}(t)$). This same argument also applies to the local perturbation that

$$g({{\rm{\Lambda }}}_{m})-g({{\rm{\Lambda }}}_{m-1})$$

also depends on the signal *q*_*m*−1_(*t*) which is stochastic in nature due to the noises added in the previous *m* − 1 segments. In that case, the stochastic random noise and the deterministic dispersion and nonlinearities will couple with each other. Furthermore,

$$g({{\rm{\Lambda }}}_{m})-g({{\rm{\Lambda }}}_{m-1})$$

will become correlated for different *m*. Our approximation decouples the two effects, resulting in a simpler model. Through the approximation, we also break the correlation among local perturbations, making channel analysis more manageable.

### Validation

In the following, we will validate Eq. (16) through numerical simulation and experiment. We investigate the perturbation of discrete eigenvalues of a 2-soliton input signal, *q*_0_(*t*) = 2*sech*(*t*), which has two discrete eigenvalues at 0.5 *j* and 1.5 *j*. Specifically, the function *g*(⋅) is a vector valued function, corresponding to the imaginary parts of the two discrete eigenvalues of the signals propagating along the fibre. First, using numerical simulation (details of simulation method are given in 5.1), we illustrate that the perturbation ${\varepsilon }_{m}=g({\hat{{\rm{\Lambda }}}}_{m})-g({{\rm{\Lambda }}}_{0})$ is signal dependent (i.e., depending on ${\bar{q}}_{m}(t)$).

**Figure 6:**
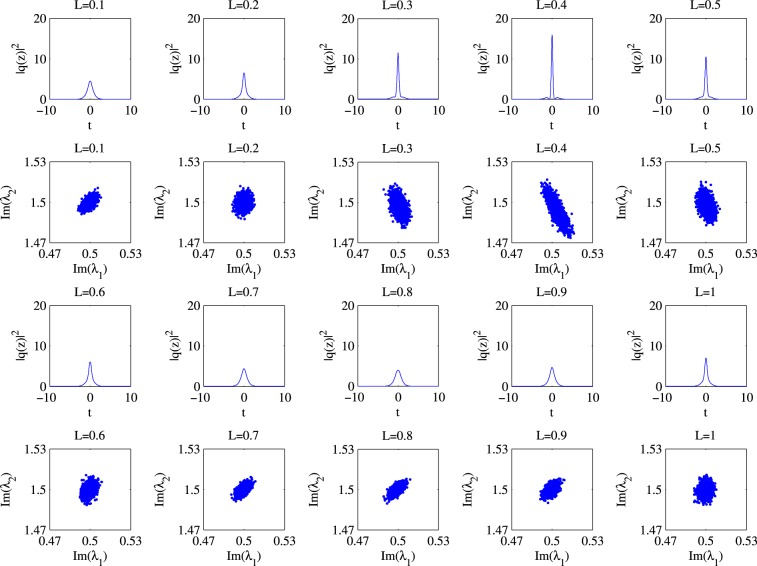
Scattering plot for ${\hat{{\rm{\Lambda }}}}_{{m}}$ at different normalized propagation lengths: 0.1, 0.2, 0.3, …, 0.9, 1.0.

We plot the ensemble of two discrete eigenvalues of ${\hat{{\rm{\Lambda }}}}_{m}$ for various *m*. Since the time domain signal will change its shape as it propagates, our numerical example clearly shows that not only the two eigenvalues are all correlated, but how they correlate depend on *m* as well (See Fig. 6). This supports our observation that the statistics of $g({\hat{{\rm{\Lambda }}}}_{m})$ (and hence also $g({\hat{{\rm{\Lambda }}}}_{m})-g({{\rm{\Lambda }}}_{0})$ as *g*(Λ_0_) is a constant) depends on ${\bar{q}}_{m}(t)$. It also suggests that the statistics of *g*(Λ_*m*_) − *g*(Λ_*m*−1_) will also depend on *q*_*m*−1_(*t*) which, strictly speaking, will also be influenced by the noises injected in the previous segments.

Observing the scattering plots for ${\hat{{\rm{\Lambda }}}}_{m}$, the mean and covariance matrix of ${\hat{{\rm{\Lambda }}}}_{m}$ depends on *m* (and more precisely, on *q*_*m*−1_(*t*)). Clearly, it also depends on the noise power. In order to better understand the influence of *q*_*m*−1_(*t*) on ${\hat{{\rm{\Lambda }}}}_{m}$, we will focus on the eigenvectors (in particular the principal one) of the covariance matrices.

Now, consider a 2 soliton *q*_0_(*t*) = 2*sech*(*t*), which has two discrete eigenvalues at 0.5 *j* and 1.5 *j*. Then ${\bar{q}}_{m}(t)$, obtained by propagating *q*_0_(*t*) for *m* segments, are also 2-solitons, with discrete eigenvalues at 0.5 *j* and 1.5 *j*. Let ${Q}_{m}^{(d)}(0.5\,j)$ and ${Q}_{m}^{(d)}(1.5\,j)$ be its corresponding spectral amplitudes. Then they will vary as *m* increases (i.e., as the signal propagates).

To make it more precise, if the normalised distance of each fibre segment is Δ_*L*_, then *q*_*m*_(*t*) is the signal after propagating *q*_0_(*t*) for a distance of *m*Δ_*L*_. If we define *θ*(*m*Δ_*L*_) as the ratio ${Q}_{m}^{(d)}(0.5\,j)/{Q}_{m}^{(d)}(1.5\,j)$, then

$$\theta (m{{\rm{\Delta }}}_{L})={e}^{4m{{\rm{\Delta }}}_{L}({\lambda }_{1}^{2}-{\lambda }_{2}^{2})j}$$

where *λ*_1_ = 0.5 *j* and *λ*_2_ = 1.5 *j*. We call $4m{{\rm{\Delta }}}_{L}({\lambda }_{1}^{2}-{\lambda }_{2}^{2})$ the *nonlinear phase difference*.

In our example, we focus on the imaginary parts of the two discrete eigenvalues. Figure 6 plots the two discrete eigenvalues, which shows that they are correlated with each other. Also, the correlation is different for different *m*. For each scattering plot, we can estimate the covariance matrix between the imaginary parts of the two discrete eigenvalues. We can also plot the angle of the principal eigenvector of the covariance matrix. Here, the principal eigenvector of a covariance matrix is the matrix’s eigenvector (often of unit length) with respect to the largest eigenvalue. In the case of a 2 × 2 matrix, we can further represent the vector by the angle it makes with the horizontal axis. It turns out that the principal eigenvector of the covariance matrix for ${\hat{{\rm{\Lambda }}}}_{m}$ depends only on the nonlinear phase.

**Figure 7:**
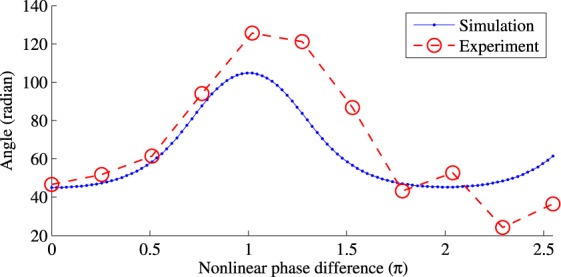
NFT phase difference between Λ_1_ and Λ_2_.

Simulation results are shown in Fig. 7, which clearly indicate that the principal eigenvector, and also the covariance matrix for ${\hat{{\rm{\Lambda }}}}_{m}$ are different for different *m*. Note that in the simulation, a noise is added to ${\bar{q}}_{m}(t)$, which is a result of propagating *q*_0_(*t*) noiselessly for a distance of *m*Δ_*L*_.

To verify our observation and to support the simulation result, we also consider the following experiment. Our experiment set up is very similar to the one in Fig. 1. EDFAs are used to amplify signals to combat signal loss. We consider a range of propagation distance up to 1500 km which consisting of 30 loops, where every 3 loops correspond to a 0.1 normalised length. This is to mimic the generation of ${\bar{q}}_{m}(t)$. Figure 7 shows the experimental values for the orientation of principal eigenvector of the covariance matrix (red circles). A qualitative agreement is observed between experiment and simulation results. This supports the assumption that eigenvalue distribution depends on signal at that point.

Note that, in experiments, noises are added at the transmitter, the receiver and also during propagation. However, the propagation noises is small (compared to the other noises), since the propagation distances are all small in our experiments. In addition, the transmitter noise is the same in all the experiments. Therefore, the difference in the distribution of eigenvalues observed at the output for different propagation lengths is only due to the receiver noise. In our experiment, the receiver noise acts as the role of the point noise injected in the simulation. Careful examination of the simulation and experiment results in Fig. 7, shows a good agreement when the propagation distance is small. However, the two results start to become less agreeable for longer distances. This can also be explained by the fact that the propagation noise increases for longer propagation distances. More discussion is available in Supplementary Material 2.

It is important to mention that the nonlinear phase difference is not the only variable that determines the angle of the principal eigenvector of the covariance matrix. Even when the nonlinear phase difference is zero, the angle is a still function of *λ*_1_ and *λ*_2_. This is evidenced by the results shown in Supplementary Material 1. Further studies are required to fully describe the relationship between the nonlinear phase and the parameters of the signal in the nonlinear Fourier domain.

So far, we have demonstrated that the local perturbation caused by the injection of noise in a segment is not identically distributed and depends on the pulse shape of the input at each segment. Next, we want to show that the overall perturbation *g*(Λ_*M*_) − *g*(Λ_0_) can be approximated by accumulating individual smaller perturbations *ε*_*m*_. In other words, we want to show that the approximation (16) is indeed fairly accurate.

**Figure 8:**
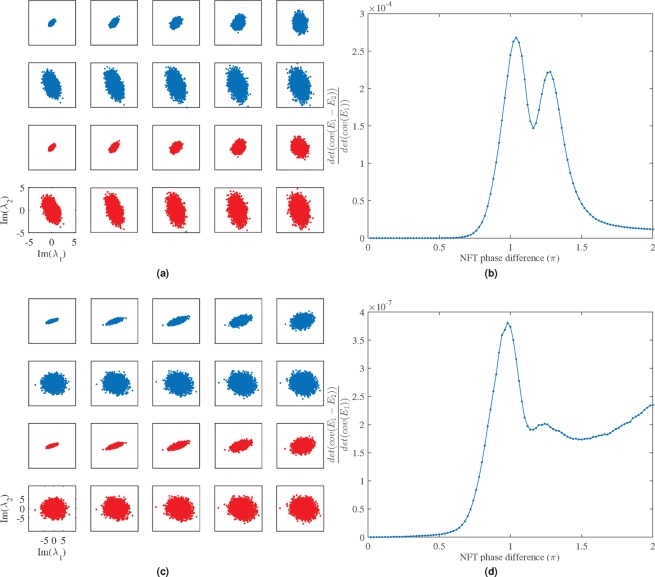
Comparison of the accumulated perturbations with (top blue) and without (bottom red) the approximation Eq. (16) for *m* values ranging from 0.2*π* to 2*π* with a step of 0.2*π*. (**a**) 2*sech*(*t*) pulses, (**b**) relative error of the approximation with 2*sech*(*t*) pulses, (**c**) square pulses with width of 10 and height of 0.5, (**d**) relative error of the approximation with the square pulses.

To validate, we will consider the following numerical example. We consider the special case when *g*_0_(*t*) is a 2-soliton. In particular, we are interested in the imaginary parts of the two discrete eigenvalues of the input and output signals. In other words

$$g({\rm{\Lambda }})={[Im({\lambda }_{1}),Im({\lambda }_{2})]}^{{\rm{T}}}$$

where Λ = (*λ*_1_,*λ*_2_). Here, we consider various choices of *m* (corresponding to NFT phase difference from 0.2*π* to 2*π*). Results are shown in Fig. 8 for 2*sech*(*t*) pulses as well as for square pulses as a demonstration for the generality of this model.

Let ${{\rm{\Lambda }}}_{m}\triangleq ({\lambda }_{m\mathrm{,1}},{\lambda }_{m\mathrm{,2}})$ be the discrete eigenvalues of *q*_*m*_(*t*), the signal obtained by propagating the input signal across *m* fibre segments. The lower scatter plot (formed by the red circles) in Fig. 8(a,c) is obtained by plotting *Im*(*λ*_*m*,1_) against *Im*(*λ*_*m*,2_) for various *m*. The upper scatter plot (formed by the blue circles) in Fig. 8(a,c) is obtained using the approximation (16), defined as the sum of *g*(Λ_0_) and a set of local perturbations caused by the addition of noises added throughout each segment. Specifically, the RHS of (16) is

$$[\begin{array}{c}0.5+Im({\sum }_{\ell \mathrm{=1}}^{m}({\hat{\lambda }}_{\ell \mathrm{,1}}-0.5j))\\ 1.5+Im({\sum }_{\ell \mathrm{=1}}^{m}({\hat{\lambda }}_{\ell \mathrm{,2}}-1.5j))\end{array}]$$

where ${\hat{{\rm{\Lambda }}}}_{\ell }=({\hat{\lambda }}_{\ell ,1},{\hat{\lambda }}_{\ell ,2})$ is the discrete eigenvalues of ${\hat{q}}_{\ell }(t)$ obtained by propagating the input signal *q*_0_(*t*) for $\ell$ segments followed by the addition of a local noise ${n}_{\ell }(t)$. Now, if we compare the two sets of scatter plots, we can see immediately that the two plots look extremely similar. A figure of merit *F* can be defined to quantify the differences, where

$$F=\frac{{\det }{(}{cov}({E}_{1}-{E}_{2}))}{{\det }{(}{cov}({E}_{1}))},$$

and

$${E}_{1}=I{m}({\lambda }_{1})-I{m}(\overline{{\lambda }_{1}}),$$

$${E}_{2}=I{m}({\lambda }_{2})-I{m}(\overline{{\lambda }_{2}}).$$

For both cases, 2*sech*(*t*) pulses and square pulses, a maximum *F* can be found around *π* NFT phase different whilst for 2*sech*(*t*) pulses, the maximum *F* is about 2.7 × 10^−4^ and for the square pulses, the maximum *F* is about 3.9 × 10^−7^. These results indicate that one can model the eigenvalues perturbation for Λ_*m*_ pretty accurately, by using our approximation.

**Remark**: The validity of the model (over the range of parameters, e.g., propagation distance) depends on several factors such as the fibre segment size and the noise power added in each segment. Generally speaking, the model will be more accurate if the length of each segment and the noise power are sufficiently small.

Specifically, there are two aspects to our model. First, we model the eigenvalue perturbation as the sum of individual perturbation introduced in each segment (due to noise). The validity of our model (or the range of the fibre length in which the model is valid) depends on the segment size. There is a trade-off between the model accuracy and the complexity (the smaller the segment size, the more complicated it is to evaluate the model).

On the other hand, we also propose to simplify the model by assuming that the perturbations introduced in each segment are independent. In this case, the validity of the model will depend on the amount of noise added in each segment (and accumulated during propagation). If the noise power is too high, then the model will become invalid.

### Simplification 2: Noise decomposition

In the previous section, we proposed a simple model to characterise the eigenvalues perturbation by modelling the perturbation separately in each segment. The noise added in each segment can be decomposed as the sum of many independent “noise components”. Depending on the decomposition, a noise component can be the noises added to a specific narrow frequency band or a time interval. In our earlier work^48^, we have demonstrated that noises added in frequency bands outside the signal frequency band will have minimal impacts on the perturbation of eigenvalues. In other words, in the context of detecting the eigenvalues, out of band noises are essentially irrelevant. Now, the natural question thus is: *Which “noise component” will contribute the most to the perturbation of eigenvalues?* A complete answer to the question remains unknown. In this paper, we will focus on specific noise components and investigate its contributions to eigenvalue perturbation.

We are interested in the noises of the same form as the input signal. We assume the input to the segment is *q*(*t*) and *n*(*t*) is the white Gaussian noise added in the segment. Define *n*_1_(*t*) and *n*_2_(*t*) such that 1) *n*(*t*) = *n*_1_(*t*) + *n*_2_(*t*), 2) *n*_1_(*t*) and *n*_2_(*t*) are orthogonal to each other, and 3) *n*_1_ is a scalar multiple of *q*(*t*). Notice that the power of *n*_1_(*t*) is significantly smaller than *n*_2_(*t*). We will call *n*_2_(*t*) the residual noise and *n*_1_(*t*) the *scaling noise*–adding *n*_1_(*t*) to *q*(*t*) is equivalent to multiplying *q*(*t*) by a scaling factor. In the following numerical example, we will evaluate the impact of scaling noise and residual noise on discrete eigenvalues.

**Figure 9:**
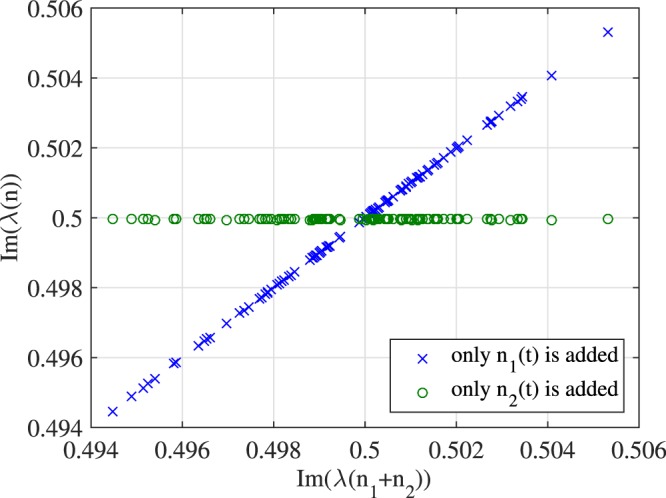
Eigenvalue perturbation errors when *n*_1_(*t*) and *n*_2_(*t*) are added separately.

**Figure 10:**
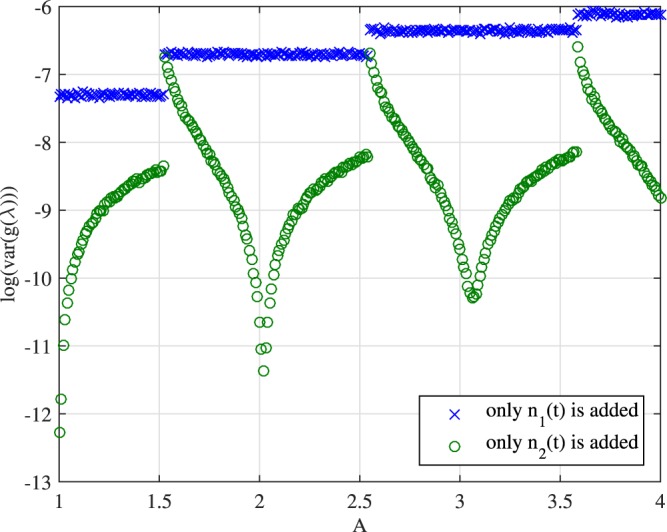
Variances of eigenvalue perturbation errors when *n*_1_(*t*) and *n*_2_(*t*) are added separately.

First, we let the signal *q*(*t*) be a fundamental soliton. And we will compare the imaginary part of the discrete eigenvalues of *q*(*t*) + *n*_1_(*t*), *q*(*t*) + *n*_2_(*t*) and *q*(*t*) + *n*_1_(*t*) + *n*_2_(*t*). Results are shown in Figs 9 and 10. In Fig. 9, we consider *q*(*t*) as the fundamental soliton first. The *x*-axis denotes the imaginary part of the discrete eigenvalue of *q*(*t*) + *n*_1_(*t*) + *n*_2_(*t*) and the *y*-axis corresponds to that of *q*(*t*) + *n*_1_(*t*) and *q*(*t*) + *n*_2_(*t*). The figure clearly shows that when only *n*_2_(*t*) is added to the signal, the eigenvalue is largely unchanged (see the green circles data points). On the other hand, when only *n*_1_(*t*) is added, the eigenvalue is essentially the same as the one obtained by adding both noises together. This example illustrates that the scaling noise is dominating the perturbation of discrete eigenvalues.

Next, we consider the case when *q*(*t*) = *A*sech(*t*). In this case, *q*(*t*) has at least two eigenvalues *A* − 0.5 and *A* − 1.5 for *A* > 1.5. In our example, we focus only on the sum of the imaginary parts of the discrete eigenvalues (which can be interpreted as the amount of energy in the solitonic component of the signal). Our simulation shows that the scaling noise *n*_1_ has a more significant impact on the perturbation (measured by variances) of the sum of eigenvalues. Specifically, we notice that

1. Eigenvalue perturbations caused by addition of *n*_1_ are often much bigger than that by addition of *n*_2_;
2. Impact caused by *n*_2_ on eigenvalue perturbation is at the smallest when *A* is close to an integer (i.e., when *q*(*t*) is a multi-soliton) and is at the largest when *A* is slightly greater than *A* − 0.5 is slightly bigger than an integer (i.e., when *q*(*t*) has an eigenvalue close to zero)
3. Impact caused by *n*_1_(*t*) is constant over regimes when *q*(*t*) has the same number of discrete eigenvalues.

Motivated by our observation, we propose the following simplification: According to our previous model, eigenvalues perturbation can be approximated by the sum of a collection of local perturbations $g({\hat{{\rm{\Lambda }}}}_{m})-g({{\rm{\Lambda }}}_{0})$ where ${\hat{{\rm{\Lambda }}}}_{m}$ are discrete eigenvalues of ${\hat{q}}_{m}(t)\triangleq {\bar{q}}_{m}(t)+{n}_{m}(t)$.

Let ${n}_{m}^{\mathrm{(1)}}(t)$ be the scaling noise component of *n*_*m*_(*t*),

$${\hat{\hat{q}}}_{m}(t)\triangleq {\bar{q}}_{m}(t)+{n}_{m}^{(1)}(t)$$

and ${\hat{\hat{{\rm{\Lambda }}}}}_{m}$ be its corresponding set of discrete eigenvalues. Then we can approximate ${\hat{{\rm{\Lambda }}}}_{m}$ with ${\hat{\hat{{\rm{\Lambda }}}}}_{m}$.

**Remark**: The merit of the simplification is that it is analytically simpler as ${n}_{m}^{\mathrm{(1)}}(t)$ is a one-dimensional noise with finite power.

**Figure 11:**
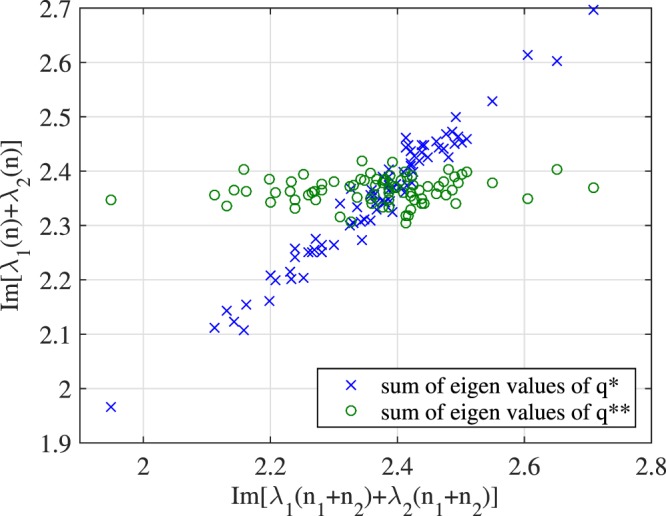
Noise decomposition for 2-soliton with discrete eigenvalues 0.9 *j* and 1.5 *j*.

Experimental observations confirm the above claim. Consider a 2-soliton input signal with discrete eigenvalues 0.9 *j* and 1.5 *j*. Figure 11 plots the sum of the imaginary parts of the two eigenvalues of *q*_*M*_(*t*), q^*^ and q^**^ as defined by; (1) *q*_*M*_(*t*) = *q*_0_(*t*) + *n*_1_(*t*) + *n*_2_(*t*), (2) q^*^(*t*) = *q*_0_(*t*) + *n*_1_(*t*) and (2) q^**^(*t*) = *q*_0_(*t*) + *n*_2_(*t*). In Fig. 11, the *x*-axis denotes the (imaginary part) of the sum of the eigenvalues of *q*_*M*_(*t*), while the *y*-axis denotes that of q^*^ and q^**^. It is observed that the perturbation of eigenvalues of *q*_*M*_(*t*) is largely contributed by the noise *n*_1_(*t*).

We want to highlight that *n*(*t*) is the additive white noise (which theoretically has an infinite power for any nonzero noise spectral density) added in a segment, and is decomposed into the sum of two noise “components” *n*_1_(*t*) and *n*_2_(*t*). Mathematically, *n*_1_(*t*) is the noise component obtained by projecting *n*(*t*) onto the signal space spanned by the transmitted signal, while *n*_2_(*t*) is the “residual” noise (i.e., the difference between *n*(*t*) and *n*_1_(*t*)). In terms of power, *n*_1_(*t*) in fact has a much lower power compared to *n*_2_(*t*). More specifically, the power of *n*_2_(*t*) will in fact scale linearity with the noise bandwidth (and hence will be large when the noise bandwidth is large), while the power of *n*_1_(*t*) stays the same. Realizing that the power of the scaling noise *n*_1_(*t*) is much smaller than the residual noise, the observation that eigenvalue perturbation is dominated by the scaling noise is unexpected.

As a final remark, we would like to point out that one of the main challenges in analysing the performance of NFT transmission is due to the inability to characterize the effect of noises. While our proposed analytical model cannot completely solve the problem, it is a first-step and offers a framework to achieve this long-term goal. Via the framework, we have been able to identify that eigenvalue perturbation can be accurately approximated as an accumulation of independent perturbations. The independence assumption in the model potentially can simplify further analysis.

## Methods

### Simulation

Numerical NFT was implemented based on the work in^6^. Forward difference method has been selected. This method recursively calculates eigenvector *ν* from initial condition (8). The time interval of the input signal [*T*_1_, *T*_2_] is divided into *N* steps with each step size (*T*_2_ − *T*_1_)/*N*. The initial condition is:

17 $$\nu ({T}_{1},\lambda )=(\begin{array}{c}1\\ 0\end{array}){e}^{-j\lambda {T}_{1}}.$$

Once the final value i.e. eigenvector *ν*(*T*_2_, *λ*) is found by recursive processing it is inserted in following equations to find *a*(*λ*) and *b*(*λ*):

18 $$a(\lambda )=\mathop{\mathrm{lim}}\limits_{t\to \infty }{\nu }_{1}(t,\lambda ){e}^{j\lambda t}$$

19 $$b(\lambda )=\mathop{\mathrm{lim}}\limits_{t\to \infty }{\nu }_{2}(t,\lambda ){e}^{-j\lambda t}$$

where $$\nu ({T}_{2},\lambda )\triangleq {({\nu }_{1}({T}_{2},\lambda ),{\nu }_{2}({T}_{2},\lambda ))}^{T}$$.

For the discrete spectrum, it is required to find the values of *λ* for which *a*(*λ*) becomes zero. This procedure was performed by creating a user-defined function in MATLAB that takes initial guess value of *λ* and uses standard functions in MATLAB to find all *λ*s corresponding to *a*(*λ*) = 0. MATLAB calls that user-defined function each time until it becomes zero.

The NFT code developed using the forward difference method was put to the test to find eigenvalues of multiple functions, e.g. fundamental soliton, non-fundamental solitons, arbitrary signals and then was checked for error percentage. The error percentage was negligibly small 10^−3^ when the number of steps *N* > 400. The implemented code was checked against a few typical pulses with known eigenvalues.

Numerical Nonlinear Pulse Propagation (NPP) based on split-step Fourier method has been widely used for simulations of nonlinear optical processes in waveguides. It is proven to have high accuracy in predicting pulse evolution during propagation. We used an in-house NPP software, written in C/C++, and used CUDA to parallelise the calculations by utilising the power of graphic processing unit (GPU). This has reduced the software run-time significantly, allowing simulating a large number of instances required for statistical evaluation. In our NPP, the propagation of a pulse along a fibre is divided into length segments within which, the nonlinear and linear processes can be separated as an approximation. In each step, a band-limited white Gaussian noise is added in the frequency domain across the whole spectrum. To limit the noise bandwidth to a certain value, we have used a bandpass filter in the frequency domain. In order to accurately describe the statistics of the model, the same propagation simulation has been repeated for 5000 times, and eigenvalues were calculated using our NFT code.

### Experiment

#### Transmission setup

The drive signals were converted to the analog domain by high-speed AWG (Keysight M8196A) operating at up to 92 GSa/sec. The lasers (both carrier and LO) used in the experiments external cavity lasers (ECL) emitting near 1,550 nm with a linewidth of ~100 kHz. The modulators used were Mach-Zehnder I/Q modulators based on LiNbO3 waveguides. A 50 km Non-zero dispersion-shifted fiber (NZ-DSF) with a nonlinear Kerr coefficient $\sim \,1.2{W}^{-1}k{m}^{-1}$, a dispersion coefficient of $\sim 4ps\cdot n{m}^{-1}k{m}^{-1}$, and 9.5 dB insertion losses was chosen as the transmission medium in the fiber loop. In this case, the distance of 150 km in a normalised NLS was around 0.1, which was obtained through the variable conversion shown in^3^. Before launched into the fibre loop, the launch powers were carefully controlled by the attenuator after a fixed gain EDFA (with noise figure 5 dB) to the optimum value. One extra EDFA was used to compensate for the remaining loss in the loop, and a flat-top optical filter with a 3 dB bandwidth of 1 nm is followed to suppress the out-of-band amplified spontaneous emission(ASE) noise. At the receiver, a polarisation controller is used to align the optical signal in the x-polarization. Then the signal was detected by a dual polarisation optical coherent receiver consists of 90 hybrids and 4 balanced pin-photodetector with 3 dB bandwidth of ~38 GHz. The output 4 E-fields waveforms were sampled by a digital storage scope (Agilent 96204Q) with a sampling rate of 80GS/s and a bandwidth of 33 GHz and stored to process offline.

#### Transmitter and receiver DSP

At the transmitter, various 2-soliton pulses were recursively computed using the Darboux transformation method^1^. The initialisation coefficients *A*_*i*_ and *B*_*i*_ for Darboux transformation method which (the discrete-spectral amplitudes and the shape of the signal) were specially chosen to get smaller physical bandwidth to improve the performance at transmitter^2^. The receiver DSP firstly used a training symbol to perform timing synchronisation. Then a pilot tone from y-polarization was used to estimate and compensate the laser phase noise and frequency offsets. After normalised by a scaling factor according to the lossless path averaged model, the synchronised pulse train was processed per pulse to search the corresponding roots. Ablowitz-Ladik algorithm was used to calculate the Nonlinear Fourier Coefficients, followed by a Newton-Raphson method for root searching^5^.

## Further Works and Conclusions

This paper focuses on perturbations/noises of eigenvalues when the optical signal is transmitted along a fibre. We have numerically and experimentally demonstrated that the noises are correlated. By exploiting the correlation, one can design a better signal constellation leading to higher system throughput. In order to take advantage of the correlation, it becomes important to derive a model of eigenvalue noises. In the second part of the paper, we have proposed an analytical framework to characterise the noises. The idea is to decouple the eigenvalue perturbation as an accumulation of many smaller perturbations, each of which is caused by the addition of noises in a short fibre segment. As a result, one can derive an eigenvalue perturbation model by characterising each smaller perturbations. Strictly speaking, all of these small perturbations are non-identically distributed and are also correlated with each other. However, we observe that the correlation is indeed quite weak that one can essentially assume them to be independent. Following the independence relation, the perturbation in eigenvalue caused by propagation can be modelled as the accumulation of a set of independently added noise.

So far, our focus is on the eigenvalue perturbation caused by noises during signal propagation. However, our modelling framework can also be extended to included noised introduced at the transmitter and the receiver. Specifically, we will model that an additive transmitter/receiver noise will be added respectively before and after the transmission. When the transmitter and receiver noises are introduced, the signal’s eigenvalues will also be perturbed. Following our paradigm, we can model the perturbations introduced at the transmitter and receiver as independent noises. Furthermore, we can also extend our work to model the perturbation of spectral amplitudes. Some preliminary correlation studies were done and can be found in^49^. In this current paper, our focus is on the perturbation of the eigenvalues. However, the same principle will also apply to spectral amplitudes as well where the overall noises in spectral amplitudes will be modelled as the accumulation of many independently distributed noises caused by the injection of noises in a short fibre segment.

## Supplementary information

supplementary materials
