# Supplementary material for: Correlated Eigenvalues of Multi-Soliton Optical Communications
Source: Sci Rep. 2019 Apr 25;9:6399. doi: 10.1038/s41598-019-42510-5 (PMC6483980; doi:10.1038/s41598-019-42510-5)
Supplement: Supplementary file 1 — supplementary materials [file 41598_2019_42510_MOESM1_ESM.pdf]

# Correlated Eigenvalues of Multi-Soliton Optical Communications: supplementary material

**Wen Qi Zhang[1], Tao Gui [2], Qun Zhang[3], Chao Lu[4], Tanya M. Monro[1,5], Terence H. Chan[†3], Alan Pak Tao Lau[2], and Shahraam Afshar V.[1,5]**

[1]Laser Physics and Photonic Devices Laboratories, School of Engineering, University of South Australia, Australia
[2]Photonics Research Center, Department of Electrical Engineering, The Hong Kong Polytechnic University
[3]Institute for Telecommunications Research, University of South Australia, Australia
[4]Department of Electronic and Information Engineering, Photonics Research Center, The Hong Kong Polytechnic University
[5]Institute for Photonics and Advanced Sensing, School of Physical Sciences, The University of Adelaide, Australia
[†]Corresponding author: Terence Chan hlchan6@gmail.com

## ABSTRACT

This document provides supplementary material to support the journal submission titled ”Correlated Eigenvalues of Multi-Soliton Optical Communications”.

## 1 Effect of different noise on eigenvalue distribution

In order to investigate the effects of noise at different stages of pulse generation collectively, we consider the most general form of noise (which can be various kinds of amplitude, phase and background noise) as follows:

$$[A_0 + A(t)]\, q(t) e^{2\pi * i * [B_0 + B(t)]} + C_0 + C(t), \tag{1}$$

where $A_0, A(t), C_0, C(t)$ are complex, and $B_0$ and $B(t)$ are real. We study the effect of different types of noise by allowing noise in each of $A_0, A(t), C_0, C(t), B_0$ and $B(t)$ separately. The following figures 1 show the NFT eigenvalue distributions for the 2-eigenvalue pulses (the same eigenvalue sets as the system described in Fig. 2 of the main document) when noise is added to each of the coefficients individually. All figures, except Fig. 1(e), represent similar trend; positive correlation and elliptical shape distribution with minor and major axes whose values and orientations depend on the values of the set of ($\lambda_1$ and $\lambda_2$). Note that for each group of simulation/experiment, a set of 2-dimensional data points (whose coordinates are the discrete eigenvalues of the received signals) will be generated. These data points further define an empirical covariance matrix. Its orientation is defined as the slope of the principal eigenvector of the covariance matrix. It is also observed that comparatively constant noises have less impact on the orientation, values of minor and major axes, and shifting of the mean values of each set than those of time-varying noises. Among time-varying noises, the phase noise, Fig. 1(f) has the most impact in terms of shifting the mean values of each set. Note that constant phase noise, on the contrary, does not affect the distribution of eigenvalues whatsoever. This agrees with analysis where it is well known that constant phase changes have no impact on discrete eigenvalues (but only on the corresponding spectral amplitudes). Although the exact contribution each type of noise is unknown, the difference between the experimental observation and simulation can be attributed to these types of noise.

## 2 Transmitter and propagation noise

An experiment was conducted to demonstrate that perturbation of discrete eigenvalues caused by the addition of point noise is input dependent (or more precisely, depends on the NFT phase of the pulse in that example). In the experiment, transmitter noise was not considered affecting the outcome, and the propagation noise in the fibre is less significant than the receiver noise. To support this assumption, Fig.2 and Fig.3 are produced. Fig.2 is a numerical simulation of a collection of pulses with noise added prior to a noiseless propagation for a distance corresponding to $\pi$ NFT phase (maximum change in the orientation of the covariance matrix). The results indicate the covariance matrix of the pulses after propagation (red circles) is the same as the pulses before propagation (blue dots). In other words, the noise generated in the transmitter does not affect the experiment.

Fig. 3 shows the comparison of the covariance matrix of the experimental datasets for 0 km and 150 km of fibre propagation respectively. The variances along the primary axis are 2.48e-3 for 0 km and 2.28e-3 for 150 km. The covariances are 1.16e-3

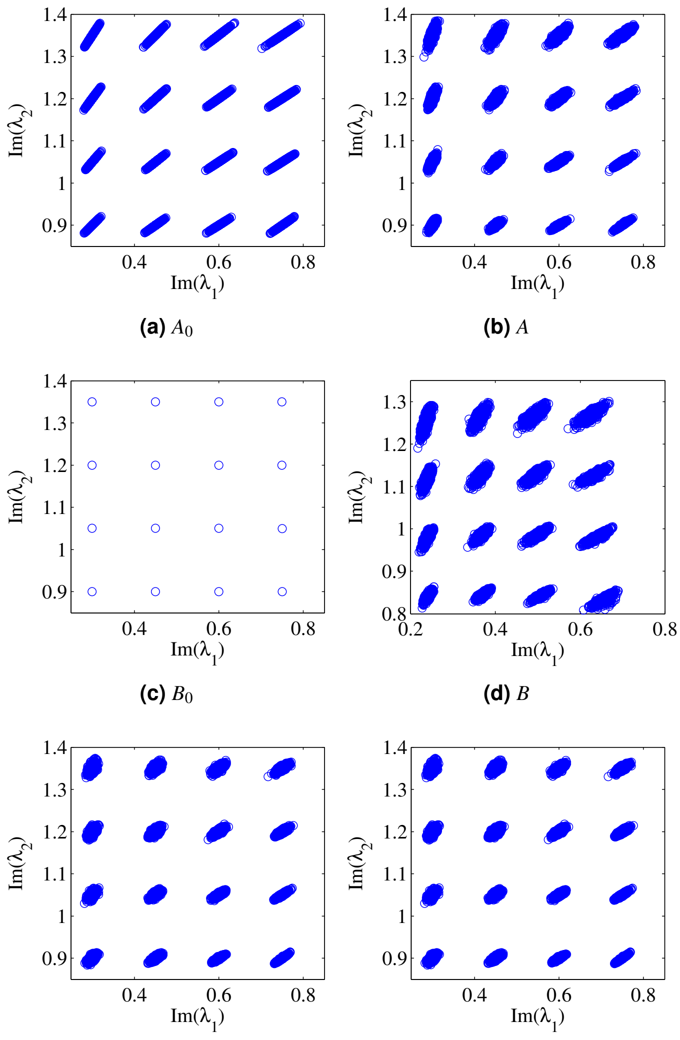


**(a)** $A_0$ **(b)** $A$ **(c)** $B_0$ **(d)** $B$ **(e)** $C_0$ **(f)** $C$

**Figure 1.** The effect of different types of noises on NFT eigen values.

for 0 km and 1.01e-3 for 150 km. We consider this amount of change in the variances to be small and thus noise add during the propagation through the fibre is small compared to the noise in the transmitter and the receiver.

In summary, the transmitter noise does not contribute to the changes in the covariance matrix of the received signals. The noise added to the signals during propagation is relatively small. Therefore, the observed rotation of the primary axis of the covariance matrix at different propagation length is mainly due to the noises added at the receiver. Hence, the experiment is equivalent to a point noise added to pulses with different NFT phases.

To see this, first, all the transmitter noise added at the input of the fibre are the same (as they all have the same input pulses). Second, as eigenvalues are invariant under the noiseless propagation, the perturbation of eigenvalues we see at the end of the fibre caused by the transmitter noise will also be the same as the input. In other words, the noise at the output due to the transmitting stage will be the same for all experiment.

## 3 Channel noise modelling via linearisation

In this paper, we proposed a method to model perturbation eigenvalues. As an analogy, our approach is, in fact, similar to the idea of approximating a nonlinear function with a piece-wise linear function via the approximation that

$$f(x_o+\delta_1+\delta_2)-f(x_o)\approx f(x_{gn}+\delta_1)-f(x_o)+f(x_o+\delta_2)-f(x_o) \tag{2}$$

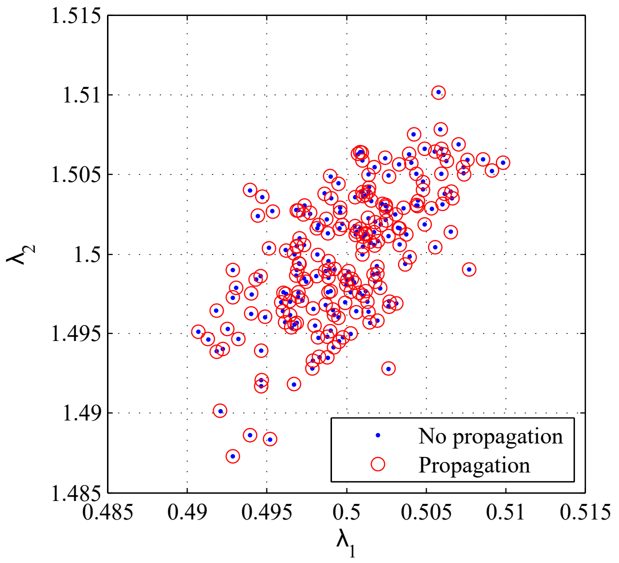


**Figure 2.** Eigenvalue perturbations caused by transmitter noise.

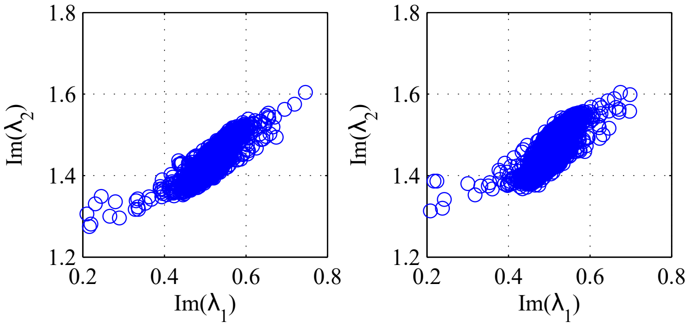


**Figure 3.** Eigenvalue perturbations in experiment. (Left) Back to back pulses. (Right) Pulses after 150 km propagation.

In other words, the error in the function $f$ caused by the noises $\delta_1$ and $\delta_2$ (either added simultaneously or sequentially) is roughly equal to the sum of errors induced by $\delta_1$ and $\delta_2$ separately. In the following, we will further elaborate our results in details.

Our first step is to verify if similar "linearity" in (2) also holds or not in our application. Consider the following simple scenario. Let $q_0(t)$ be an input signal and $\Lambda_0 = (\lambda_{0,i}, i \in |\Lambda_0|)$ be its corresponding set of discrete eigenvalues and $g(\Lambda_0)$ be a function of $\Lambda_0$ of interest. Note that $g(\cdot)$ can be a vector-valued function in general. The following are some examples:

$$g(\Lambda_0) \triangleq \min_{i=1,\ldots,|\Lambda_0|} \text{imag}(\lambda_{0,i}), \tag{3}$$

$$g(\Lambda_0) \triangleq \max_{i=1,\ldots,|\Lambda_0|} \text{imag}(\lambda_{0,i}), \tag{4}$$

or

$$g(\lambda_0) \triangleq \sum_{i=1,\ldots,|\Lambda_0|} \text{imag}(\lambda_{0,i}). \tag{5}$$

Now, consider two noises $n_1(t)$ and $n_2(t)$. Let

$$q_1(t) = q_0(t) + n_1(t)$$
$$q_2(t) = q_0(t) + n_2(t)$$
$$q_3(t) = q_0(t) + n_1(t) + n_2(t).$$

The addition of noises will cause the discrete eigenvalue to perturb. Specifically, let $\Lambda_k = (\lambda_{k,i}, i \in |\Lambda_k|)$ be the eigenvalues of $q_k$ respectively for $k = 1,2,3$. Now, the question is to determine whether

$$g(\Lambda_3) - g(\Lambda_0) \approx g(\Lambda_1) - g(\Lambda_0) + g(\Lambda_2) - g(\Lambda_0) \tag{6}$$

holds or not.

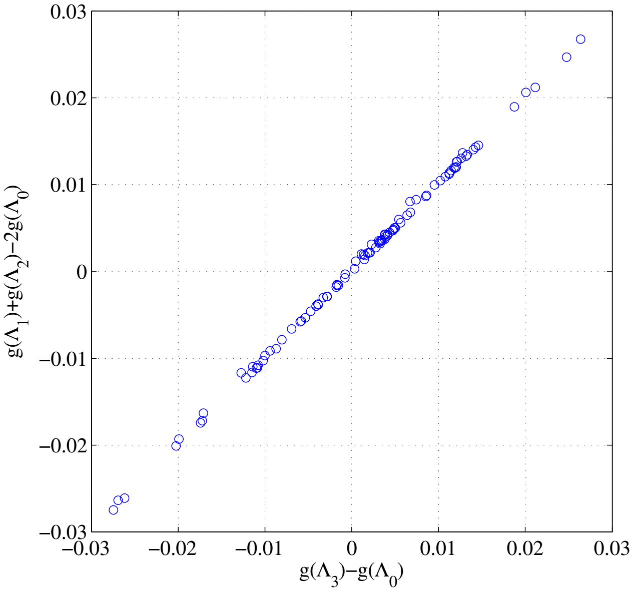


**Figure 4.** Eigenvalue perturbations add up linearly.

To answer the question, we conduct the following numerical example, in which two Gaussian noises ($n_1(t)$ and $n_2(t)$) are added to a 2-soliton $q_0(t)$. We consider a scalar-valued function $g$ as defined in (5), which can be interpreted as the energy of the signal's solitonic components. Results are shown in Figure 4 where the $x$-axis corresponds to $g(\Lambda_3) - g(\Lambda_0)$ and the $y$-axis corresponds to $g(\Lambda_1) + g(\Lambda_2) - 2g(\Lambda_0)$. Our numerical example clearly indicates that the two quantities are basically the same suggesting that "linearity" in (6) does hold. The previous example suggests that the errors/perturbations on the eigenvalues are in fact linear – that the accumulated errors induced by the addition of two noises are approximately the sum of the two errors induced by the noises separately. Next, we will consider the case when the noises are added in a distributed manner along the fibre.

## 4 An illustration of improving data rate using correlated egienvalues

Consider input signal constellation of 2-solitons pulses, with eigenvalues $\lambda_1 \in [0.7j,\ 0.74j,\ 0.78j,\ 0.82j]$ and $\lambda_2 \in [0.9j,\ 0.92j,\ ...,\ 1.00j,\ 1.02j]$. For each input, we propagate the signal in fibre for a normalised distance of 0.1. Figure 5(a), shows the distribution of the two eigenvalues for the output pulses, clearly showing the correlation between the eigenvalues. Now, suppose the correlation is ignored. Then the decoder will assume in the worst case that the two eigenvalues are independently distributed. Figure 5(b) shows the distribution of two eigenvalues assuming that they are in fact independently distributed. This leads to the requirement that the eigenvalues of input signals should be separated sufficiently enough to ensure that the decode can distinguish different inputs with high accuracy. On the other hand, by exploiting correlations, we can pack more inputs with eigenvalues in range of $[0.7j, 0.82j]$ and $[0.9j, 1.02j]$ such that the decoder can still decode with low error probability. Figure 5(c) shows a 10 fold larger constellation, using the same range of eigenvalues for $\lambda_1$ and $\lambda_2$, but denser packing of the eigenvalues of input signals. In this example, the data rate has been increased from $\log 24$ bits per symbol to $\log 84$ bits per symbol. Clearly, the actual increase in data rate will depend on a lot of other factors, such as power and bandwidth constraint.

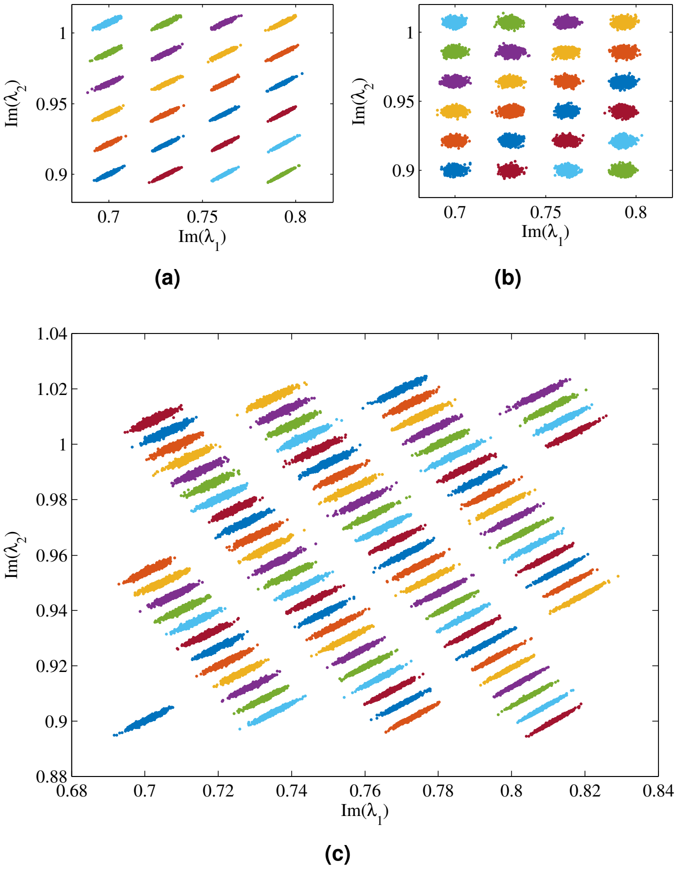


**Figure 5.** Distribution of eigenvalues for constellation of 2-soliton pulses. (a) distribution of eigenvalues for a $4 \times 6 = 24$ constellation. (b) distribution of eigenvalues for a $4 \times 6$ constellation but assuming no correlation. (c) distribution of eigenvalues for a 83 constellation.
